# The Development of Spatial–Temporal, Probability, and Covariation Information to Infer Continuous Causal Processes

**DOI:** 10.3389/fpsyg.2021.525195

**Published:** 2021-03-05

**Authors:** Selma Dündar-Coecke, Andrew Tolmie, Anne Schlottmann

**Affiliations:** ^1^Centre for Educational Neuroscience and Department of Psychology and Human Development, UCL Institute of Education, University College London, London, United Kingdom; ^2^Department of Experimental Psychology, University College London, London, United Kingdom

**Keywords:** probability, covariation, spatial–temporal thinking, causation, causal processes, development

## Abstract

This paper considers how 5- to 11-year-olds’ verbal reasoning about the causality underlying extended, dynamic natural processes links to various facets of their statistical thinking. Such continuous processes typically do not provide perceptually distinct causes and effect, and previous work suggests that spatial–temporal analysis, the ability to analyze spatial configurations that change over time, is a crucial predictor of reasoning about causal mechanism in such situations. Work in the Humean tradition to causality has long emphasized on the importance of statistical thinking for inferring causal links between distinct cause and effect events, but here we assess whether this is also viable for causal thinking about continuous processes. Controlling for verbal and non-verbal ability, two studies (*N* = 107; *N* = 124) administered a battery of covariation, probability, spatial–temporal, and causal measures. Results indicated that spatial–temporal analysis was the best predictor of causal thinking across both studies, but statistical thinking supported and informed spatial–temporal analysis: covariation assessment potentially assists with the identification of variables, while simple probability judgment potentially assists with thinking about unseen mechanisms. We conclude that the ability to find out patterns in data is even more widely important for causal analysis than commonly assumed, from childhood, having a role to play not just when causally linking already distinct events but also when analyzing the causal process underlying extended dynamic events without perceptually distinct components.

## Introduction

Hume (1739/1978) argued that we can only know about causality from the “constant conjunction” of potential causes and effects. Since then, multiple schools of thought have put some form of statistical analysis of repeated experience at the core of causal thinking, ranging from the causal attribution literature in social psychology (Kelley, 1967, 1973) to work on associative causal learning inspired by animal studies (Shanks and Dickinson, 1988). However, although causes covary with their effects, inferring causation from correlation has many pitfalls. Kantian reasoning instead focuses on the underlying causal mechanisms that allow causes to generate their effects, and modern approaches attempt to integrate such mechanism-based thinking with statistical analyses (see Waldmann, 2017).

When given a choice, people tend to seek information about mechanisms (how a process works) rather than covariation (inferring joint variability of two random variables) to determine causality (Ahn et al., 1995). People seem to recognize that statistical information needs to fit with the mechanism, because it is the latter that generates the covariation of cause and effect. However, in many situations, the underlying generative mechanism is unknown. In such cases, statistical reasoning, forms of analyses based on information about the frequency of occurrence or co-occurrence of potential causes and effects, is crucial for causal thinking (e.g., Cheng, 1997; Griffiths and Tenenbaum, 2009).

Analyses of statistical regularities between events presuppose that there are separate events to be linked into cause-and-effect sequences, for instance, when pushing a button is followed by a light coming on or when a ball is set in motion by collision with another ball. Most studies of causal thinking have considered causal sequences with such distinct components.

We do, however, also reason about causality in naturally continuous processes, without clear segmentation into potential cause and effect, as when an object sinks, for instance, or dissolves in water. The observation here is of continuous change, and while we may think about what causes this change, or what causes one of its features, for instance, why one object sinks slow, while another sinks fast, in our perceptual experience, the process has no naturally distinct components to serve as candidate cause and effects. One can nevertheless focus, in thought, on aspects of the process and think about the underlying causal mechanism, of course, but it is not so clear anymore whether and how statistical reasoning contributes to causal reasoning here.

We have recently begun to study children’s causal thinking in these types of dynamic natural processes, taking an individual difference approach and finding that measures of what we call spatial–temporal analysis were important predictors of children’s thinking about the causal mechanisms involved (Dündar-Coecke et al., 2019, 2020). Spatial–temporal analysis is the ability to think about how spatial configurations change over time. It is separate from children’s verbal and non-verbal IQ and from their spatial ability, which is not predictive of causal thinking. Spatial–temporal analysis goes beyond purely spatial analysis in that it includes the ability to work out the temporal order of a series of spatial states and the ability to project these state transformations onto past, present, and future experiences. Spatial–temporal analysis might thus help children find segmentations for continuous processes, which in turn would be helpful for causal reasoning about them.

In the present paper, we use a similar individual difference approach to return to the more Humean question of whether aspects of children’s statistical thinking also predict their causal reasoning about continuous processes, and how such statistical predictors compare to their spatial–temporal predictors. We present further data from the project reported in Dündar-Coecke et al. (2019, 2020), which developed a set of novel tasks to look at children’s causal thinking about continuous processes (sinking, absorption, and dissolving). It also involved a large battery of spatial–temporal, spatial, verbal, and non-verbal reasoning tasks, as previously reported, and in addition, the set of statistical reasoning tasks that are the focus of the present paper. In subsequent sections, we discuss in more detail our statistical thinking measures and their possible links to causal thinking.

## On the Link Between Probabilistic Thinking and Causal Processes

Probabilistic reasoning enables one to estimate of the likelihood of an event that may or may not occur (mud suggests rain). In a world where causal processes are induced by complex set of factors, it is crucial to analyze the degree of certainty of causal relationships because in most circumstances there are unobserved latent factors, which allow exceptions (not all mud suggests rain, but sometimes flooding). In some circumstances, probabilistic thinking can be used as a tool to improve the accuracy of our decisions even in the absence of mechanism knowledge.

Interest in the role of probability has already led to psychological investigation. In Piaget and Inhelder (1975) studies, the development of such thinking was seen as a formal operational achievement. The emphasis in this approach was on improvements in children’s ability to quantify the relative proportions of target and non-target events as they get older. A more recent approach, in contrast, has focused on children’s intuitive understanding. Multiple studies have shown that children’s probability judgments conform to the structure of normative probability concepts, e.g., taking an appropriate ratio from kindergarten age (e.g., Anderson and Schlottmann, 1991; Schlottmann and Anderson, 1994; Acredolo et al., 1989; Schlottmann, 2001, reviewed in Schlottmann and Wilkening, 2012). Even younger, pre-school children already have a basic ability to discriminate predictable from unpredictable event sequence (Kuzmak and Gelman, 1986), and there have been multiple demonstrations in recent years that infants have some sensitivity to different sampling processes (Xu and Denison, 2009). Thus, early capabilities of engaging in rudimentary probability calculations co-exist with difficult tasks that are computationally challenging for young children and have high demand on memory skills (e.g., White, 2014; McCormack et al., 2015, 2016).

These demonstrations involve elaborate lengthy experimental tasks that would not be suitable for a correlational study. Here, we use the abbreviated versions of the probability tasks and investigate whether probabilistic thinking is relevant to reasoning of continuous causal processes. We hypothesize that children’s ability to judge probability may not just index computational ability, but also sensitivity to definiteness of outcomes in the world. To test this hypothesis, we first observe children’s sensitivity to probability along with their computational abilities. Further, we investigate whether the development of probability understanding is linked to children’s reasoning about continuous causal phenomena (sinking, absorption, and solution). Third, we compare these competences with children’s performances on spatial–temporal measures. The predictive tasks – probability, covariation, spatial–temporal – were presented to elicit whether children’s computational ability or sensitivity to probability mattered for the inference of causal processes. This three-stage investigation helps us to identify how individual differences in such probability judgments might link with individual differences in reasoning about temporally extended processes above/beyond other reasoning types.

The tasks in which children exhibit these abilities typically involve non-causal models, displaying all outcome possibilities simultaneously to minimize memory requirements. For example, in the first probability task (marbles), the child sees a plate with seven red winner marbles and three blue loser marbles and judges how easy it is to win in a blind draw. In tasks where probabilities are experienced sequentially (e.g., the child draws a number of times from a population with initially unknown proportion of winner and loser marbles), children do not do so well when predicting the next outcome, as has long been known from work on probability learning (Brainerd, 1981) and child variants of the Iowa Gambling Task (Huizenga et al., 2007). Children’s difficulties in sequential tasks may reflect memory capacity and other processing limitations, though, and in any case indicate problems with cumulative estimation rather than basic grasp of probability [these two types of tasks address different aspects of understanding, as discussed in Schlottmann and Wilkening’s (2012) review].

The marbles task captures children’s sensitivity to probability rather than their computational ability. It derives appreciation of uncertainty and likelihood from rational analysis that multiple outcomes are possible in a given situation and from enumeration of these outcomes, prior to experiencing instances of the outcomes themselves. Probability tasks laying out all outcome possibilities simultaneously for children (e.g., showing them all the marbles on a plate) provide opportunity for such analysis. Children typically do well on these.

Another probability task derived probability from sampling – a distribution of variable outcomes over time, which ostensibly requires greater attention to the detail of that distribution, where frequencies of outcomes needed to be observed over many trials. Sequential probability tasks are modeled on this, conforming to the way in which probability is often encountered in everyday life, where we may not have an *a priori* idea of the likelihood of an outcome, or indeed even of the fact that the outcome is variable, until we begin to experience the situation. Even though children do not do so well on these tasks, due to higher processing demands, these skills still link to probability understanding (Bayless and Schlottmann, 2010).

Probability understanding *per se* comes prior to the ability to calculate probabilities, which is largely established in early years (Acredolo et al., 1989; Bryant and Nunes, 2012). Children’s understanding of how to quantify it may be restricted to simple relations like “more” or “larger,” as Bryant and Nunes showed in their large-scale intervention that more refined proportional reasoning is highly trainable regardless of children’s initial ability and that training is effective during the elementary years, indicating that it too is within children’s competence in this age range.

A task with lower computational demand -appropriate for the age range- was needed. Therefore, the ‘randomness’ task was added to the battery to address the fact that sometimes outcomes are determined and predictable, while in other situations they may be unpredictable or potentially random (Reyna and Brainerd, 1994; Bryant and Nunes, 2012). Children seem to make this distinction from ages 4 or 5, as shown by Kuzmak and Gelman (1986), who presented children two devices: one deterministic (marbles lined up in a clear tube, with the first coming out on each trial) and one a lottery device (a cage full of spinning marbles). Children understood that in the first device each outcome is known, but in the second, it is not. Study 1 here employed a similar task, the distribution of target cards in shuffled and unshuffled decks, with an anticipation that this would be sensitive even to the youngest children’s abilities.

Altogether, this study included three probability tasks, with different levels of processing complexity. These tasks may elicit variation in performance at different ages and clarify which task might be related to which aspect of thinking about continuous causal processes, such as relative “definiteness” of effect (e.g., stones are very likely to sink, berries and grapes are less likely to) or, as noted earlier, unobservable causal mechanisms (i.e., some other factor affects the relative probability of sinking).

## On the Link Between Covariation Information and Causal Processes

Grasping bivariate distributions may be more demanding than univariate distributions, because children must track variation in not just one, but two variables, and recognize whether this indicates a link between them. In probability tasks, instead, children need to evaluate the likeness of an event, where the ratio varies between impossibility and certainty. Detection of such links would clearly be helpful in identifying potentially causal variables. For instance, in Schulz et al.’s (2008) study, preschoolers were shown pairs of gears (B and C) operating with a causal chain and a common cause structure on the basis of observing interventions between them. Children as early as 4 years old could discriminate between causal chain and common cause structures (see also Shultz and Mendelson, 1975; Shultz, 1982; Schulz et al., 2008; Sobel et al., 2009).

Considering the Humean regularity and Kantian generative mechanism approaches, Schulz (1982) worked with 3- to 13-year-olds. He reported five experiments, where, for instance, sound, wind, and light transmissions were presented to children in different procedures to assess the essential meaning of causation for children. Children received problems on each of these apparatuses: transmission from source, temporal contiguity versus generative transmission, spatial contiguity versus generative transmission, and covariation. Similar to Ahn et al.’s (1995) findings, he found that children consistently prefer generative mechanism rather than covariation information when they see a conflict between them. For instance, children’s justifications were mostly based on mechanism, but rarely based on covariation, even when 3-year-olds’ verbal abilities were poorer than the elders at the generative aspects of the problem. However, contrary to Ahn et al.’s (1995) proposal, Schultz’s results showed that the tendency to analyze causal mechanism is not restricted to prior knowledge – whether children were familiar with the objects or with transmission rules (see also Koslowski et al.’s (1989), for supporting evidence with college students). These studies showed that children can grasp causal relations in the absence of probability or covariation information (see also Perales et al., 2010).

The interest is typically on whether children grasp the implications of covariation information about distinct events for causation. These studies mostly compare the simple case of two potential causes, one regular and one irregular covariate of the effect. To reduce processing demands, only minimal information is given, on whether a cause always co-occurs with the effect (AB cases), or whether in some instances a cause occurs without the effect (A not B cases). If both frequencies are considered, one can derive the probability of the effect, given the cause. This, however, is only part of true covariation assessment, which also requires consideration of the base rate, the conditional probability of the effect occurring in the absence of the cause (i.e., not AB versus not A not B cases) in terms of a 2 × 2 contingency table, as shown in Table 1.

**TABLE 1:** A typical 2 × 2 contingency table, with cause and effect as the two variables.

	Blossomed	Dead
Plant received fertilizer	AB	A not B
Plant did not receive fertilizer	Not AB	Not A not B

The literature focused on covariation (or contingency) judgment therefore considers how humans utilize information from all four cells. A well-established approach is based on the delta *p* statistic (Jenkins and Ward, 1965; Dennis and Ahn, 2001; Marsh and Ahn, 2009), which is the difference between the two probabilities discussed above (the probability of the effect given a cause and the conditional probability of the effect occurring in the absence of the cause). Adult covariation judgment is often studied by providing numerical summaries of the instances in explicit contingency tables, though the instances can, of course, also be presented sequentially, as in the real world, which adds memory demands. To avoid this, and also lower the numerical requirements of such tasks, pictorial formats are typically used with children (see, e.g., Shaklee and Mims, 1981). Note that, as in Table 1, these types of studies still illustrate covariation information in causal contexts, to attempt to make complex structured data patterns intuitive and meaningful for children.

Even so, however, children commonly fail to use the delta *p* strategy appropriately, but instead employ simpler strategies that do not consider all four cells of the table or do not weight them evenly. Using this approach, Shaklee and Mims (1981) demonstrated four strategies used by children across development, hierarchically increasing – from the least to the most sophisticated: judgment of the frequency with which the target events co-occur (AB), comparison of the number of times target events do and do not co-occur (AB versus A not B), comparing frequencies of events confirming and disconfirming the relationship (AB plus not A not B versus A not B plus not AB), and optimal assessment of the difference between two conditional probabilities (delta *p*).

These patterns suggest a shift from less to more accurate use of covariation data, where frequency judgment based simply on positive co-occurrence emerges early, while the conditional probability strategy does not appear until the 10th grade. Consistent with this, Shaklee and Paszek (1985) found that, in elementary school, children were most likely to make judgments about covariation by comparing frequencies of the target event and the use of the more advanced strategies identified by Shaklee and Mims (1981) was rare even in fourth grade. Similarly, Ferguson et al.’s (1984) data showed that older children’s impressions were influenced more by fuller covariation information rather than frequency information *per se*. In this study, 5- to 13-year-olds were presented with three scenarios about a boy displaying harmful behavior. In condition 1, the harm-doing behavior was low in consistency and also low in frequency. In condition 2, the harm was high in consistency and also high in frequency. In the third condition, the harm was low in consistency but high in frequency. Even preschoolers showed the sensitivity to the frequencies and to the stability of the boy’s behavior, but the use of covariation information increased clearly with age.

We hypothesize that primary age children’s apparent tendency to focus on frequency over covariation may reflect their difficulties of understanding, but it may also be influenced to some extent by the tasks used. When computational demands, such as ratios and percentages, are minimized, even young children appreciate the difference between variables that co-vary perfectly with an effect or are unrelated to it. For instance, Schulz et al.’s (2008) experimental design with four conditions showed that children can clearly observe a block hitting another block causing it to emit either a train or siren noise. Assessment of imperfect correlation poses more problems, though this is affected too by the way information is presented. For instance, in simple symmetrical tasks (asking whether green or red chewing gum causes bad teeth as illustrated over 10 pictures), even 4-year-olds could evaluate patterns of covariation (Koerber et al., 2005).

**FIGURE 1:**
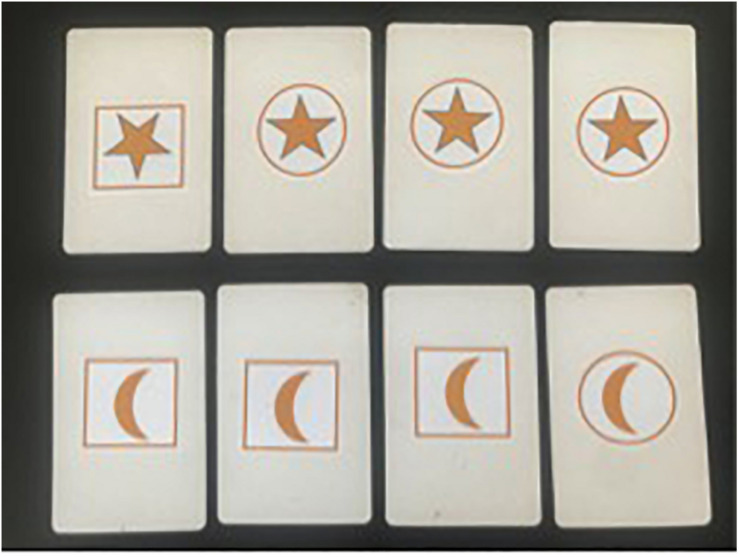
One of the four trials of the covariation task employed in Study 2, displaying the first deck with 75% co-occurrence between stars and circles, representing imperfect covariation.

To test this hypothesis, we devised a non-causal covariation task to assess whether individual differences in covariation assessment predict children’s causal thinking. We kept the task as simple as possible, using a pictorial approach, consistent with the literature, and with our other tasks, we investigated children’s assessment of simple covariation patterns. The task included four decks, each consisted eight cards, in which a particular surround shape (a circle or square) contained a particular symbol inside (a star or a moon). Attention focused throughout the degree of co-occurrence between stars and circles. As shown in Figure 1, in the first deck, the co-occurrence between stars and circles was 75%. In the second deck, co-occurrence was 50% (analogical to A not B cases). In the third, it was 100% (AB cases). For each deck, children were requested to answer verbally whether a star went together with a circle. Further, they were asked to evaluate how likely a star went with a circle. To answer this question, children were presented with a paper showing a line starting from “never go together” to “always go together.” For each deck, children ticked on this line where they think the likelihood would be best represented. They were encouraged to answer the question by thinking with percentages as well. The focus of this task was on whether children could extract this relation in a number of problems differing in the relative frequency of co-occurrence. We elicited simple verbal and non-verbal responses, but did not ask children to explain their responses, or question them about more complex data.

Overall, in the present study, we employed five probability and covariation tasks, aiming to obtain reliable measures of individual variation in children’s statistical thinking to map onto variation in causal inference. Children had to assess frequency relationships *per se*, not frequency relations between cause and effect.

## Study 1

Study 1 tested the above hypotheses by working with 5- to 11-year-old children. The study employed three causal tasks in relation to continuous processes, one spatial–temporal ability task, one covariation and three probability tasks, and measures of verbal and non-verbal ability as controls.

### Methods

#### Design

The study utilized a combined cross-sectional and individual differences design, employing three groups spanning the English primary (elementary) school age range. We focus here on 10 tasks that were given to children in fixed order within a single one-to-one session: measures of verbal and non-verbal ability, three mini-experiments focusing on causal thinking, and a spatial–temporal task, plus the three probability and covariation tasks.

One-way ANOVAs were used to test for differences between age groups on each task. Fitness of the regression models initially tested by looking at linear, logarithmic, and quadratic trends. Pearson and partial correlations (controlling for age) showed the strength of the associations between the measures. Estimates of the unique variance explained by each predictor task in causal measures were tested using hierarchical linear regressions. Adjusted *R*^2^ values showed the variances explained by the final models. Possible confounds in these estimates were checked with mediation analyses. Combined patterns were tested using path analysis.

#### Participants

The sample comprised of 107 children, recruited with parental consent from schools in London and Oxford: 35 of them from year 1 (Y1, *M*_age_ = 6.1 years, sd = 4.4 months), 33 from year 3 (Y3, *M*_ag__e_ = 8.4 years, sd = 5.9 months), and 39 from year 5 (Y5, *M*_age_ = 10.3 years, sd = 5.9 months). The sample encompassed wide ethnic and linguistic variation but was skewed toward the upper range in terms of socioeconomic background.

#### Materials and Procedure

Testing took place out of class in a quiet area within school and, for the tasks described here, lasted on average 35 min per child. Responses were recorded manually on score sheets, but children’s replies during the causal tasks were also audio-recorded.

*The causal tasks* were developed by the authors for this particular project and focused in turn on two contrasting instances of sinking (a stone and a grape sinking), absorption (a piece of tissue and blotting paper absorbing water), and solution (rock and table salt dissolving in water). Comparison between these instances revealed differences, as one item sank slow, another fast, which may then be linked to concurrent differences and commonalities between the objects (e.g., the stone is heavier than the grape, but they are of similar size), which would not be salient in an individual instance.

The tasks were administered and scored as described in Dündar-Coecke et al. (2019). Children were asked to predict outcomes ahead of witnessing simultaneous demonstration of the two instances, which they were then asked to describe, and to explain, as a measure of causal inference assessing the identification of basic factors, operative variables, and mechanisms. Two types of measure were computed from these tasks: totals for accurate prediction from prior knowledge and description for each of the instances considered (maximum = 6) and for inference (ascending score for level of response for each task; maximum = 9); and a total score for causal performance across these indices (alpha = 0.751), which could range from 0 to 21. Interest centers here on the overall causal measure and the measure of inference as the key component where sensitivity to probability and covariation might be anticipated to have an influence.

Appendices 1, 2 provide the full details of task administration and scoring. To confirm reliability, two authors subsequently scored all responses independently from the audio-recordings. Agreement rate was 93%, and final scores were assigned following discussion and checking the audios in the small number of instances where there was a difference. Examples for response levels can be seen in Supplementary Table 13 in Supplementary Appendix 2.

##### Measure of spatial–temporal analysis

**FIGURE 2:**
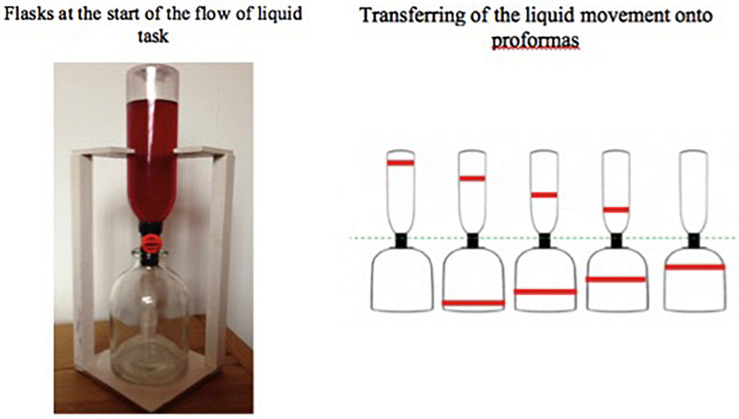
Flasks at the start of the flow of liquid task.

The *flow of liquid* (FOL) task, adapted from Piaget (1969/2006), examined children’s ability to analyze the FOL from one container to another at successive time points and to reconstruct the sequence of change. It consisted of three stages. At the first, two flasks were presented one on top of the other with a tap between (Figure 2). The upper flask (I) was filled with red-colored water, while the lower (II) was empty. Children were given a *pro forma* showing both flasks with a space between them, and they marked the respective levels in the flasks by drawing horizontal lines on the *pro forma*. The liquid was then allowed to flow from I to II in four further steps, and the child marked the liquid level on a fresh *pro forma* each time, being invited to correct any errors. At stage two, the five proformas were shuffled and the child put them in order, again being invited to correct any errors. At the third stage, each *pro forma* was cut in two, separating drawings of I from II, shuffled, and the child attempted to put them in order again. Children were expected to match the upper and lower bottles correctly and also put them in the right sequential order. Scores were based on the number of drawings in the correct position at this stage and could therefore range from 0 to 10.

##### Understanding of probability and covariation

The *randomness* task was used to explore children’s understanding of the consequences of a chance mechanism. Participants were shown two identical decks of 30 cards, five of which had smiley face stickers, with the remainder blank. The cards with the stickers were placed at the top of each deck, face up, so that they were visible. One of the decks was then shuffled so that the cards with smiley faces were now mixed with the blank cards. The two decks were then put face down, and participants were asked: “If you want to make sure to pick a smiley face, which deck would you pick from, and why?” Children’s choices were marked as 0 or 1 depending on whether they chose the shuffled or unshuffled deck, and if they made the correct choice, their explanations were marked as 0 or 1 according to whether they were able to identify the predictability of the position of the cards with the smiley faces as key to making a choice. Scores could range from 0 to 2.

**FIGURE 3:**
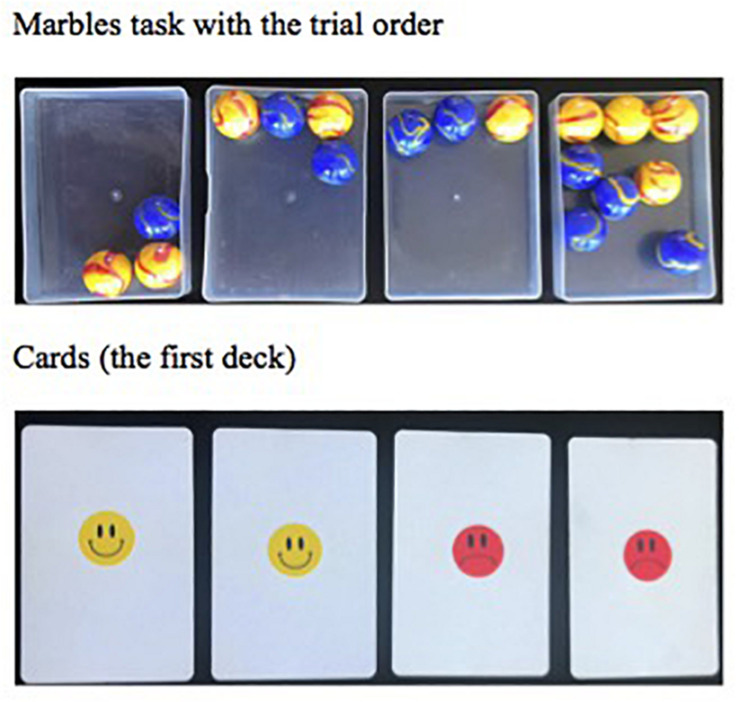
(a) The four trays for the four trials of the Marbles task, in trial order; (b) the deck shown in the first trial of the cards task – only one smiley and one frown were dealt face up, the other two cards were shown face down.

The *marbles task* was adopted from Piaget and Inhelder (1975) to evaluate children’s understanding of proportions without sampling. Children were shown over four trials four trays with different numbers of colored marbles (see Figure 3). After being told that blue marbles were the winners, children had to say how good each tray was for winning if one marble was picked with eyes closed. They were also asked to estimate how likely they would be to pick a winner from each and could express their answer verbally as either fractions/ratios (as some older children did spontaneously), or by ticking on a line from “never get one” to “always get one.” Fully correct answers on both parts of the question were scored as two points for each tray, and partially correct scored as one. Participants who gave consistent correct answers for the second and the fourth tray received an extra two points for confirming verbally that the proportions were identical. This yielded an overall score ranging from 0 to 10.

The *cards task* was developed by the authors to assess children’s understanding of frequencies based on sampling. Children saw over four trials four decks comprised of different numbers of cards with smiley versus sad face stickers: (1) two smiley, two sad (see Figure 3); (2) two smiley, four sad; (3) four smiley, two sad; and (4) four smiley, four sad, thus utilizing the same proportions as in the marbles task, to ensure that any differences in difficulty between marbles and card tasks did not just reflect differences between samples presented. On each trial, they saw half of the cards dealt out face up, selected to represent the overall proportions, with the others remaining face down. Children had to say how good each deck was for picking a smiley, and then like the marbles, estimate the chances of doing so. Scores were similar as to the marbles task and could range from 0 to 10.

The *covariation task* was developed by the authors. The task involved three trials on a laptop, each displaying, in pseudorandom order, a series of eight pictures, half in a square and half in a circle frame. Children had to detect whether there was a relation between frame shape and content of the picture.

**FIGURE 4:**
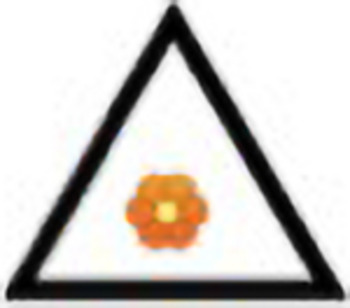
Practice display of the covariation task.

The task was started with an introduction, displaying a triangle together with a flower (Figure 4). Children were told “Now, you are going to see some shapes appear one by one on the screen filled with different pictures like this following: a flower goes with a triangle. I will ask you each time look at the screen carefully and tell me what shape goes with what picture.”

**FIGURE 5:**
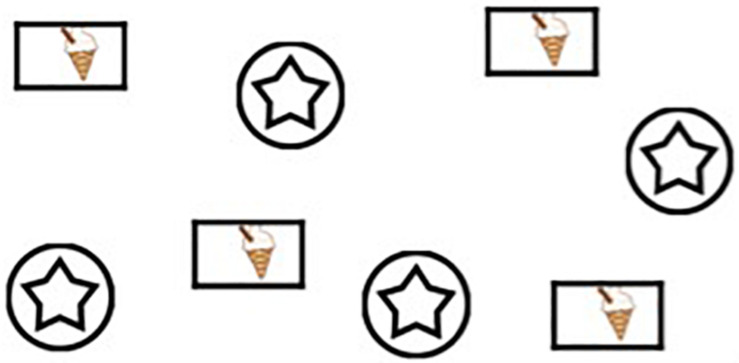
First display of covariation task representing perfect covariation.

The first display showed perfect covariation: four pictures of an ice cream in the square and four of a star in the circle (see Figure 5). Each figure appeared on the screen one by one, and children were asked: *Which shape goes with the ice cream? Do they always go together? Which shape goes with the star? Do they always go together?*

The second display showed imperfect covariation (75% contingency): three pictures of a basketball and one of sunglasses in the squares, and three of a phone and one of a line in the circle. Participants were asked: *Which picture goes with the circle? Do they always go together? Which picture goes with the square? Do they always go together?* The third display had no pattern (zero contingency), the circles and squares all contained different pictures, and participants were again asked the same questions. Shapes were kept consistent to provide a common anchor across displays, but pictures were varied, to avoid carry-over. All trials consisted of eight figures each. Co-occurrence could be expressed as fractions/ratios, or by ticking on a line, as for marbles, from “can’t tell at all” to “definitely.” Each correct answer was marked as 1 point. Children were expected to identify of the dominant correlate for displays 1 and 2, and they were supposed to say “none”/“any” for display 3 based on the appropriate estimation of the strength of association. Scores could range from 0 to 12.

##### Measures of verbal and non-verbal ability

The expressive vocabulary and block design subtests from the Wechsler Abbreviated Scale of Intelligence (WASI) (Wechsler, 2011) were used to provide standard measures of verbal and non-verbal ability.

The WASI vocabulary is a measure of expressive language, word knowledge, and verbal concept formation. Children were required to define the words when the researcher read aloud. Administration and scoring followed standard procedures.

The WASI Block Design is a subset to explore children’s non-verbal cognitive abilities. Children were shown nine red and white square blocks and a book illustrating different patterns in each page that could be made with the blocks. Children were asked to arrange the blocks to match each design shown in the picture, increasing in difficulty. This task aimed to measure children’s ability to analyze and synthesize abstract representations within specific time limits for each display.

### Results

Analyses utilized data from the 107 participants who completed testing, except where noted. Age trends on each measure are presented below, followed by analyses of relationships between the causal, spatial–temporal, probability, and covariation measures. All statistical tests were two-sided where the highest *p* value was set to 0.05. Employing the *F* and *t* test procedures based on the general linear model of regression, the observed power for the regression was 0.95, which was calculated using G^∗^Power 3.1.9.2 (Erdfelder et al., 2007).

#### Developmental Trajectories

**FIGURE 6:**
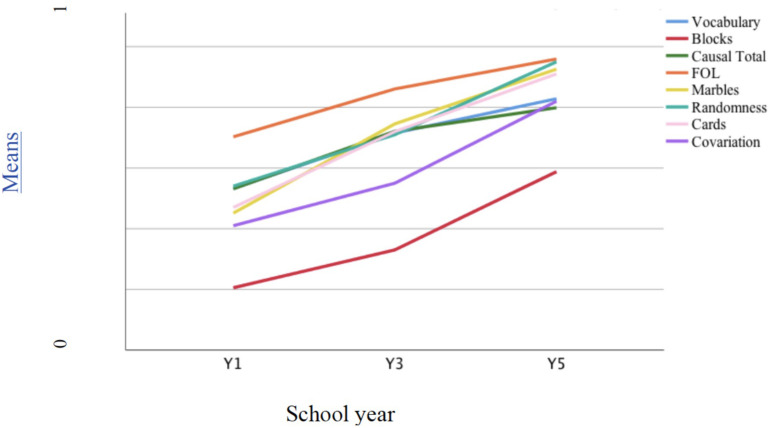
Developmental trajectories of all variables computed using standardized measures across the three year groups.

There were a significant negative skew on total causal score, FOL, randomness, marbles, and cards and a positive skew on block design, due to the youngest and oldest age groups, respectively, exhibiting a longer tail of scores; inference, covariation, and vocabulary were normally distributed. Figure 6 demonstrates the developmental trajectory for each measure using scores standardized to a scale between 0 and 1 for comparability. Overall, there was a clear upward trend for all tasks, but with variation in relative difficulty. Block design was in particular difficult for most children. Significant negative skew indicated that most children failed to gain higher scores in this task, while FOL was easier (hence the difference in direction of skew), with causal total lying in between. Comparing the trends, the steepest gradients were seen for blocks and covariation, followed by randomness, cards, and marbles.

Means and standard deviations on the original scales are shown in the Table 2 (study 1). In terms of differences between age groups, marbles, FOL, causal total, and vocabulary tasks showed a similar pattern: the steepest increase was between Y1 and Y3, with a slow down subsequently. For the rest, the steepest gradient was between Y3 and Y5, except randomness where growth was linear. One-way ANOVAs by school year found highly significant increases with age on all variables, using the Welch robust statistic, *p* < 0.001 in each case. The majority of inference responses on all three causal tasks focused solely on relevant factors or variables (scores of 1 or 2), though mechanism responses were more evident among older children: 2.9% of children in Y1 gave one or more mechanism response, 24.2% in Y3, and 30.8% in Y5 (cf. Dündar-Coecke et al., 2019, for the response profiles of the causal tasks).

**TABLE 2:** Study 1 mean scores (with standard deviations) on total causal performance (max = 21), inference (max = 9), vocabulary (max = 43), block design (max = 58), flow of liquid (FOL; max = 10), randomness (max = 2), marbles, cards (max = 10), and covariation (max = 12).

	Y1	Y3	Y5	Total
Causal total	10.63 (4.44)	14.42 (2.96)	15.97 (2.44)	13.75 (4.04)
Inference	3.34 (1.89)	5.06 (1.54)	5.54 (1.54)	4.67 (1.90)
Vocabulary	22.89 (5.29)	30.76 (5.86)	35.62 (5.20)	29.95 (7.59)
Blocks	11.91 (6.08)	19.15 (9.52)	34.10 (13.25)	22.23 (13.86)
FOL	7.03 (3.27)	8.61 (2.09)	9.59 (1.31)	8.45 (2.55)
Randomness	1.09 (0.89)	1.42 (0.79)	1.90 (0.38)	1.49 (0.78)
Marbles	4.51 (3.08)	7.45 (2.95)	9.26 (1.82)	7.15 (3.29)
Cards	4.69 (3.56)	7.15 (2.76)	9.10 (1.59)	7.06 (3.27)
Covariation	4.83 (2.18)	6.61 (3.29)	9.82 (2.81)	7.20 (3.48)

Study 2 mean scores (standard deviation) on total causal performance (max = 33), inference (max = 12), flow of liquid (FOL, max = 12), DTV (max = 18), marbles (max = 10), covariation (max = 8), block design (max = 45), and vocabulary (max = 43).				

Causal total	15.33 (4.76)	16.20 (4.47)	18.72 (2.95)	16.82 (4.31)
Inference	5.03 (2.36)	5.69 (2.56)	6.63 (1.75)	5.82 (2.32)
Vocabulary	22.48 (5.39)	29.05 (5.01)	34.01 (4.56)	28.86 (6.77)
Blocks	12.43 (5.62)	16.45 (7.12)	24.01 (8.97)	17.91 (8.78)
FOL	8.33 (4.34)	9.24 (3.79)	11.16 (2.40)	9.65 (3.72)
DTV	12.58 (3.50)	13.33 (3.08)	14.56 (2.77)	13.54 (3.18)
Marbles	3.76 (2.99)	5.39 (3.05)	7.41 (2.95)	5.61 (3.31)
Covariation	4.48 (1.96)	5.34 (2.35)	6.34 (1.96)	5.44 (2.22)

#### Relationships Between Causal Performance and Spatial–Temporal Analysis, Probability, and Covariation

##### Correlations between variables

The relationship of the predictor variables to the causal measures was linear, apart from block design, where it was logarithmic (*R*^2^ for linear fit = 0.263; while it was 0.368 for logarithmic trend). Zero-order Pearson correlations between the different measures showed overall causal performance and inference was strongly positively associated with all the potential predictors, which were themselves all positively correlated with each other (Table 3, study 1 correlations). The high correlations between causal total and inference were plausible, as causal total contained prediction, description, and inference scores across the three tasks.

**TABLE 3:** Study 1 zero-order and partial correlations between measures (zero-order correlations above diagonal, *N* = 107; partial correlations below diagonal, controlling for age in months, verbal and non-verbal ability, *N* = 106 due to missing date of birth data for one participant; significant values in bold, *p < 0.05, **p < 0.01, and ***p < 0.001).

	Causal total	Inference	Vocabulary	Log blocks	FOL	Randomness	Marbles	Cards	Covariation
Causal total	1	0.90***	0.54***	0.61***	0.52***	0.39***	0.55***	0.52***	0.56***
Prior	0.69***	0.53***	0.47***	0.56***	0.46***	0.27**	0.42***	0.42***	0.47***
Description	0.79***	0.70***	0.44***	0.48***	0.45***	0.40***	0.42***	0.49***	0.43***
Inference	0.85***	1	0.47***	0.52***	0.42***	0.34***	0.53***	0.43***	0.51***
Vocabulary	–	–	1	0.68***	0.44***	0.44***	0.52***	0.53***	0.64***
Log blocks	–	–	–	1	0.43***	0.41***	0.56***	0.54***	0.62***
FOL	0.30**	0.20*	–	–	1	0.35***	0.55***	0.56***	0.47***
Randomness	0.13	0.10	–	–	0.16	1	0.49***	0.54***	0.43***
Marbles	0.25*	0.28**	–	–	0.38***	0.28**	1	0.76***	0.63***
Cards	0.21*	0.13	–	–	0.38***	0.36***	0.61***	1	0.60***
Covariation	0.20*	0.21*	–	–	0.20*	0.15	0.37***	0.32**	1

Study 2 zero-order and partial correlations between measures (zero-order correlations above diagonal, partial correlations below diagonal, controlling for age in months, verbal and non-verbal ability, *N* = 124; significant values in bold, *p < 0.05, **p < 0.01, and ***p < 0.001).									

	**Causal total**	**Inference**	**Vocabulary**	**Log blocks**	**expFOL**	**DTV**	**Marbles**	**Covariation**	

Causal total	1	0.89***	0.53***	0.48***	0.60***	0.44***	0.39***	0.43***	
Inference	0.82***	1	0.55***	0.49***	0.61***	0.49***	0.42***	0.41***	
Vocabulary	**–**	–	1	0.53***	0.49***	0.39***	0.48***	0.43***	
Log blocks	**–**	–	0.53**	1	0.50***	0.42***	0.55***	0.34***	
expFOL	0.41***	0.41***	–	–	1	0.54***	0.52***	0.43***	
DTV	0.23*	0.30**	–	–	0.38***	1	0.44***	0.39***	
Marbles	0.10	0.14	–	–	0.29**	0.23*	1	0.40***	
Covariation	0.24**	0.20*	–	–	0.25**	0.25**	0.20*	1	

When age in months and verbal (vocabulary) and non-verbal ability (log block design) were controlled for, only FOL, marbles, cards, and covariation remained significantly associated with total causal performance. The same set of variables was also related to inference, with the exception of cards. FOL was related to both cards and marbles and to covariation to a lesser extent. The probability and covariation measures were predominantly related to each other, though marbles and cards were the most closely related measures, with covariation – and randomness – more distinct from these. Randomness had little relation to the causal measures, possibly because its narrow scoring range made it less discriminating. It did not affect the beta values of other variables and remained non-significant in each regression model and was therefore discounted from further consideration.

##### Hierarchical regression models

Hierarchical regression was used to examine the unique variance accounted for by the remaining predictors. Taking total causal score and inference in turn as the dependent variable, age in months and vocabulary were entered in the first stage of the analysis. Marbles, cards, and covariation were entered after the control variables, but with marbles first, since it related best to the causal indices; this made it possible to assess its specific impact before including cards and covariation. Log block design was entered at the fourth stage, in order to assess the influence of verbal and non-verbal ability separately and to examine the predictive power of the statistical measures before and after non-verbal ability was controlled for. The spatial–temporal measure, FOL, was entered at the fifth stage, since it appeared to be the most robust predictor overall. Analyses for prior knowledge and description with the same order of entering predictors are presented in Appendix 5.

For *total causal score*, the analysis (Table 4, study 1 regressions) produced significant Δ*R*^2^ at each stage except the third. Age and vocabulary were significant predictors at the first stage, but the beta for vocabulary dropped and age was superseded by marbles when that was entered. Vocabulary and marbles dropped out when cards and covariation were added, but neither of the latter was significant, indicating that all four predictors shared variance. The beta for cards was smaller than that for covariation, which was marginally the largest remaining predictor. Log block design was a significant predictor when added at the fourth stage, and produced further drops in the betas for all the other variables, with a bigger impact on covariation than marbles or cards. FOL joined log block design as a further predictor at the final stage, without substantially affecting the betas for the other variables, except marbles.

**TABLE 4:** Study 1 hierarchical regression analysis with *total causal* score as dependent variable (significant predictors in bold).

Model	M1	M2	M3	M4	M5	
**Predictor**	**B**					

Age in months	0.332**	0.207	0.176	0.115	0.109	
WASI vocabulary	0.310**	0.231*	0.154	0.059	0.044	
Marbles		0.310**	0.172	0.145	0.096	
Cards			0.104	0.086	0.033	
Covariation			0.184	0.131	0.121	
Log blocks				0.284*	0.273*	
Flow of liquid					0.203*	

Adj*R*^2^ = 0.454; Δ*R*^2^ = 0.347*** for M1; 0.061** for M2; 0.022 for M3; 0.035* for M4; and 0.026* for M5. *p < 0.05.**p < 0.01.***p < 0.001.						

Study 2 hierarchical regression analysis with total causal score as dependent variable (significant predictors in bold).						

**Model**	**M1**	**M2**	**M3**	**M4**	**M5**	**M6**

**Predictor**	**β**					

Age in months	−0.136	−0.171	−0.174	−0.249*	−0.230*	−0.218*
WASI vocabulary	0.624***	0.551***	0.483***	0.434***	0.408***	0.349**
Marbles		0.202*	0.147	0.050	0.017	−0.053
Covariation			0.221**	0.209*	0.176*	0.130
Log blocks				0.284**	0.250*	0.197*
DTV total					0.160	0.062
Expflow of liquid						0.349***

AdjR^2^ = 0.459; ΔR^2^ = 0.292*** for M1; 0.031* for M2; 0.037** for M3; 0.046** for M4; 0.018 for M5; and 0.066*** for M6. *p < 0.05.**p < 0.01.***p < 0.001.

The analysis for *inference* produced similar outcomes at the first two stages (Table 5, study 1 regression), except age and vocabulary that were both superseded by marbles. In this case, however, the addition of cards and covariation had little appreciable impact on marbles. Covariation had the second largest beta, but was not significant. The inclusion of log block design had little impact on marbles, cards, and covariation. The addition of FOL had somewhat more impact on marbles, but the latter remained the sole significant predictor.

**TABLE 5:** Study 1 hierarchical regression analysis with *inference* as dependent variable (significant predictors in bold).

Model	M1	M2	M3	M4	M5	
**Predictor**	**β**					

Age in months	0.285*	0.144	0.127	0.083	0.080	
WASI vocabulary	0.272*	0.182	0.126	0.060	0.050	
Marbles		0.351**	0.321*	0.301*	0.273*	
Cards			−0.068	−0.081	−0.111	
Covariation			0.190	0.153	0.147	
Log blocks				0.201	0.194	
Flow of liquid					0.118	

Adj*R*^2^ = 0.339; Δ*R*^2^ = 0.262*** for M1; 0.078** for M2; 0.017 for M3; 0.018 for M4; and 0.009 for M5. *p < 0.05.**p < 0.01.***p < 0.001.						

Study 2 hierarchical regression analysis with *inference* as dependent variable (significant predictors in bold).						

**Model**	**M1**	**M2**	**M3**	**M4**	**M5**	**M6**

**Predictor**	**β**					

Age in months	−0.174	−0.215*	−0.217*	−0.291**	−0.265**	−0.253**
WASI vocabulary	0.667***	0.583***	0.531***	0.482***	0.445***	0.392***
Marbles		0.237**	0.195*	0.100	0.053	−0.010
Covariation			0.169*	0.157	0.110	0.069
Block design (log)				0.280**	0.233*	0.184*
DTV total					0.224**	0.135
Expflow of liquid						0.317***

AdjR^2^ = 0.488; ΔR^2^ = 0.318*** for M1; 0.042** for M2; 0.022* for M3; 0.044** for M4; 0.035** for M5; and 0.054^***^ for M6. *p < 0.05.**p < 0.01.***p < 0.001.

Overall, the regression analyses revealed clear overlaps between the influence of all the predictors on causal performance. However, the relative impact of including FOL and log block design in the models indicates marbles and cards were somewhat more closely related to the former and covariation to the latter. Probability and covariation therefore appeared to capture somewhat different dimensions, in line with the partial correlations. In particular, while spatial–temporal and non-verbal ability were the strongest predictors of overall causal thinking, for inference, the effects of probability were stronger.

##### Nature of shared variances between predictors

Factor analysis with varimax rotation was used to explore in more depth the nature of the relationship between FOL, log block design, marbles, cards, and covariation, given their shared influence on the causal indices. The Kaiser–Meyer–Olkin (KMO = 0.834) measure of sampling adequacy was well within acceptable limits. The KMO identified a four-factor solution that explained 95% of the shared variance between the five measures, which confirmed separable components relating to marbles/cards, covariation, FOL, and log block design (Table 6, study 1 rotated component matrix).

**TABLE 6:** Study 1 four-factor model for flow of liquid, log blocks, marbles, cards, and covariation (significant predictors in bold).

	Factor 1	Factor 2	Factor 3	Factor 4
FOL	0.292	**0.927**	0.163	0.168
Log blocks	0.281	0.172	**0.907**	0.264
Marbles	**0.821**	0.233	0.234	0.298
Cards	**0.856**	0.257	0.223	0.208
Covariation	0.335	0.189	0.287	**0.876**

Study 2 three factor solution for exponential flow of liquid, DTV, log block design, marbles, and covariation.				

	**Factor 1**	**Factor 2**	**Factor 3**	

Exp FOL	0.461	**0.648**	0.252	
DTV total	0.191	**0.915**	0.152	
Log blocks	**0.857**	0.228	0.080	
Marbles	**0.794**	0.233	0.246	
Covariation	0.194	0.213	**0.951**	

**FIGURE 7:**
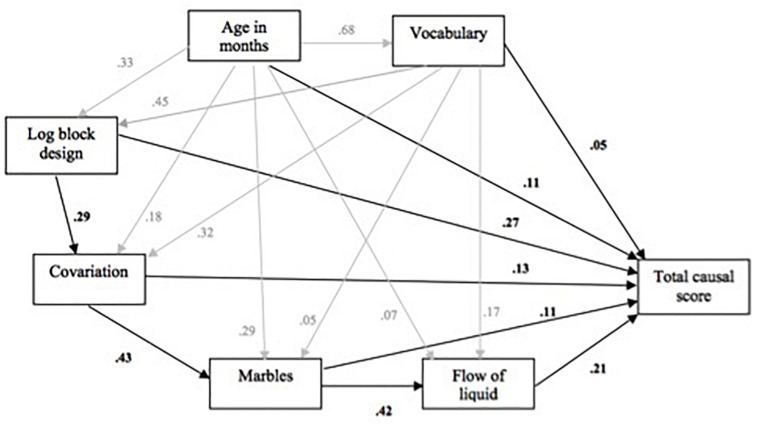
Extended mediation model for the effects of log block design, covariation, marbles and flow of liquid on total causal score (subsidiary effects in gray).

In view of this, maximum likelihood path analysis was used to examine whether there were specific directional relationships between the predictors that would explain the observed patterns of overlap in their influence on the causal indices. For both causal measures, the best fit was provided by an extended mediation model, which was assessed by the chi-squared and probability values penalized by the Akaike information criterion (AIC), where the fitness of the model improved as the AIC value lowered. The best fit was χ^2^ = 3.891, *p* = 0.273 for total causal and χ^2^ = 3.887, *p* = 0.274 for inference, with *df* = 3 for both. Figure 7 illustrates the model and path coefficients obtained for total causal score. Black and gray paths were used to distinguish between subsidiary and major path coefficients. The model illustrated a stable pattern of effects in which non-verbal ability, awareness of covariation, and probability (as indexed by marbles) support spatial–temporal analysis, but with each also influencing aspects of causal reasoning to different degrees. For overall causal performance, non-verbal and spatial–temporal ability have the largest direct effects, with the effects of probability and covariation smaller by comparison; for inference, the direct effect of probability, 0.219, is stronger than non-verbal and spatial–temporal ability, 0.190 and 0.101, respectively. Age and vocabulary have little or no direct impact on causal thinking in these models and act as background variables, influencing the main predictors to different degrees.

Further moderation analyses confirmed there were no interaction effects between log block design, marbles, covariation, or FOL in predicting causal scores.

### Discussion

This study confirmed developmental trends in the ability to analyze probability, covariation, and spatial–temporal information, with clear increases across the age groups, though the statistical indices used here showed later growth than the spatial–temporal indices, with children approaching ceiling by Y5 on FOL.

Performance on FOL remained discriminating, but the use of statistical information consistently correlated with both overall causal thinking and inferential level causal analysis. Factor analysis confirmed these are distinct competences, though classical and frequentist probability was found to be closely related. Probability, covariation, and spatial–temporal analysis had, in part, independent effects on causal inference, but also, in part, interrelated influence connected to non-verbal ability. Marbles performance was a significant predictor to begin with in both regression analyses, but for overall causal thinking, it dropped substantially with the inclusion of cards and covariation, and then again when FOL was added. For inference, however, it remained a significant predictor once included, though it was again affected by FOL. Covariation was never a significant predictor. This might be partly attributable to the limited number of steps involved in the task we used affecting its sensitivity – but it nevertheless had a sizeable beta until log block design was included, and it interacted with FOL at lower levels. Although verbal ability had no impact on any aspect of causal performance, this may have been due to the relatively narrow social range of the sample; there were nevertheless clear indications that statistical ability in particular overlapped in part with verbal ability.

The findings suggested that only spatial–temporal analysis and non-verbal form of cognitive ability significantly associated with causal thinking; neither statistical inference nor verbal ability had significant explanatory power. However, we need to consider the sample and task characteristics before arriving at conclusions. Thus, the next study refined the task battery and examined the replicability of findings among a wide range of population.

## Study 2

The modified causal tasks followed the structure of a scientific investigation, the FOL task was extended, an additional spatial–temporal measure was derived from an adaptation of Wilkening’s (1981) distance/time/velocity integration tasks, and a more socially representative sample within the same age range was employed.

The marbles task remained to assess children’s probability judgments. Another covariation task, which was a revision of that used in study 1, utilized physical materials in the form of decks of cards rather than a computer display, in keeping with most of the other test materials. The tasks were selected on the basis of their relative predictive strength in study 1. Therefore, the cards task was dropped, in view of its overlap with marbles in study 1, and randomness was dropped because of its lack of predictive power.

### Methods

#### Design

The design, age groups, task order, and administration were all equivalent to study 1. We focus on nine tasks given in fixed order within a single one-to-one session: WASI expressive vocabulary and block design (Wechsler, 2011), three causal experiments, and two spatial–temporal tasks, plus the probability and covariation tasks at the end.

#### Participants

The sample comprised 124 children, recruited with parental consent from three schools in Oxford: 36 from Y1, mean age = 5 years, 11 months, sd = 3.8 months; 45 from Y3, mean age = 7 years, 11 months, sd = 3.6 months; and 43 from Y5, mean age = 9 years, 9 months, sd = 5.1 months. Children’s ethnic and linguistic background was similar to study 1 but covered a more broadly representative range of socioeconomic backgrounds.

#### Materials and Procedure

Testing for the tasks took an average of approximately 42 min per child (min = 29, max = 57). Responses were recorded in the same way as for study 1.

The *scientific method causal tasks* were developed by the authors and administered and scored as described for study 2 in Dündar-Coecke et al. (2020). The tasks followed a more realistic scientific procedure, with a sequence of observation, description, prediction, justification, and explanation. Full details of this protocol can be found in Appendix 3. Briefly, the tasks again focused in turn on contrasting instances of sinking, absorption, and solution, but in this case, children first observed and described two instances before being asked to predict. They then justified their predictions by judging the outcomes of further three items. Children then explained the influences at work across all five instances. Two types of measure were computed from these tasks: totals of each task for accurate description (maximum = 3), prediction and justification (each maximum = 9), and level of explanation (again, assessing identification of basic factors, operative variables, relationships between variables, and mechanisms; maximum = 12). The second measure was the total score for causal performance across all indices, 0–33, alpha = 0.724. Interest again centered on the overall score for causal performance and that for inference.

Appendices 3, 4 provide the details of the scripts and scoring systems. As in the first study, children’s responses were scored independently by two authors based on the criteria shown in Supplemenatry Tables 14, 15 in Supplementary Appendix 4. The independent scores were compared for interrater reliability. Any difference in the independent scores was followed by further checking of the audio records, with a discussion to get a 100% agreement on the final scores.

The measures of *verbal and non-verbal ability* and the FOL were all similar as described for study 1, except that six stages were employed for FOL rather than five, and scores could therefore range from 0 to 12. The distance–time–velocity (DTV) measure required children to make estimates of each of distance, time, and velocity in turn, by integrating information about the other two variables. Each task utilized scenarios akin to those employed by Wilkening (1981), displayed on PowerPoint slides. For *distance*, children judged how far three animals varying in speed (cat, mouse, and turtle) would run in a fixed time, counted out by the experimenter, to escape from a barking dog. For *time*, they had to estimate, by counting themselves, how long an animal (cat, bunny, and turtle) would take to run to a fixed point, with the second half of the run concealed behind a wall. For *velocity*, they had to judge which of seven animals (deer, horse, cat, bunny, mouse turtle, and snail) would make it to a fixed destination in a given period of time, counted out by the experimenter. Children’s judgments relied entirely on mental projection based on information provided, and no actual motion was observed to support the key elements of these. Each task consisted of three trials, with responses on each trial scored 0–2 in terms of degree of accuracy. The total score across the three tasks could therefore range from 0 to 18.

##### Measure of probability

The *marbles task* followed exactly the same procedure for administration and scoring as in study 1.

##### Measure of covariation

The *covariation task* was developed by the authors to provide an alternative approach to the previous computer-based covariation task. The task utilized cards showing one of four images: a circle containing a star, a circle containing a crescent moon, a square with a star, and a square with a moon. Attention focused throughout on the degree of co-occurrence between stars and circles, with the squares as distractors. Children saw four decks in turn consisting of eight cards. In the first deck (Figure 1), three of the circles contained stars and one a moon; co-occurrence between stars and circles was therefore 75%. In the second deck, co-occurrence was 50%: half of the circles contained stars and half contained moons. In the third, it was 100%: all circles contained stars. In the fourth, it was 0%: all the circles contained moons.

In each case, the cards were laid out face up before the child in random order, and they were asked to say from what they saw in front of them how often stars and circles went together. As in the marbles task, they made a verbal judgment first of all (e.g., “three times,” “always”), and then provided an estimate of frequency by ticking on a line, one end marked “never” and the other “always.” Correct answers on both responses were scored as two points for each deck, allowing for some lack of exact precision in the tick responses. Partially correct responses were scored as one. Scores therefore varied between 0 and 8.

### Results

Analyses utilized data from all 124 participants. Age trends are presented first, followed by analyses of relationships between causal, spatial–temporal, probability, and covariation measures. All statistical tests were two-sided. The highest *p* value was set to 0.05. Using the G*Power 3.1.9.2 (Erdfelder et al., 2007), the observed power for the regression was 0.97.

#### Age Profiles

**FIGURE 8:**
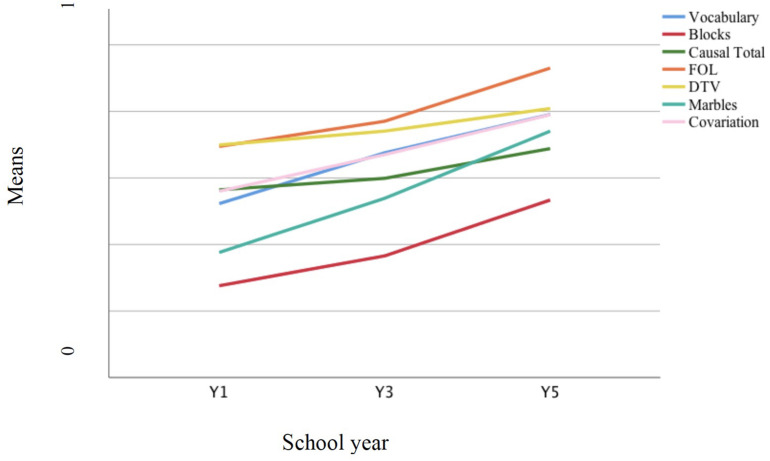
Developmental trajectories of all variables computed using standardized measures across the three year groups.

Mean scores on each measure are shown in Figure 8, using standardized measures, as in study 1. There were a significant negative skew on FOL, DTV, and vocabulary and a positive skew on block design; the remaining variables were normally distributed. Again, the developmental trend was clear, with tasks varying in difficulty. Blocks task was in particular difficult for children, while FOL was easier. The causal task was also more difficult than in study 1.

Comparing the increase in scores across year groups, the steepest gradients were apparent for blocks, FOL, and causal total, with the greatest growth between Y3 and Y5 (see Table 2, study 2 mean scores). The remaining measures showed a linear trend with marbles exhibiting the steepest gradient. One-way ANOVAs by school year found significant increases with age on all variables, using the Welch robust statistic, *p* < 0.01 in each case, except DTV, *p* < 0.05. For all measures, there were significant increases in scores from Y1 to Y5. Performance on FOL was similar to study 1, again approaching ceiling by Y5, despite the extra step in the procedure; the later growth on this and the causal indices more probably reflected the mixed sample of study 2 lagging behind the higher socioeconomic status (SES) sample of study 1. In line with this, these children exhibited marginally lower mean scores for vocabulary (albeit with lower variance) and notably lower scores on block design, marbles, and – even allowing for the change in task – covariation. Mechanism responses were also less common – 4.6% of responses in Y1 were at this level, but only 11.1% in Y3, and 17.8% in Y5.

#### Relationships Between Causal Performance and Spatial–Temporal Analysis, Probability, and Covariation

##### Correlations between variables

The fitness tests assessing the trends between the predictor and causal variables showed that block design was again logarithmically related to the causal measures [*R*^2^ for linear fit = 0.194 (*F*(1, 122) = 29.375, *p* = 0.000); *R*^2^ for logarithmic fit = 0.232 (*F*(1, 122) = 36.849, *p* = 0.000], and FOL was marginally exponential [*R*^2^ for linear fit = 0.342 (*F*(1, 122) = 63.546, *p* = 0.000); *R*^2^ for exponential fit = 0.357 (*F*(1, 122) = 67.810, *p* = 0.000]; relationships for the other predictors were linear. As in study 1, zero-order Pearson correlations showed the causal indices were strongly positively associated with all the potential predictors, and the predictors were themselves all positively correlated with each other, as shown in Table 3, study 2 correlations.

Controlling for age in months, vocabulary, and log block design, only FOL, DTV, and covariation remained significantly associated with total causal score and inference. In contrast to study 1, marbles was unrelated to either causal measure. Marbles and covariation were more weakly related than in study 1, possibly reflecting the revised measure of the latter and the lower SES sample, and there was a more equivalent relationship between each and the spatial–temporal measures; the strongest relationship was between the spatial–temporal measures, FOL, and DTV.

##### Hierarchical regression models

The predictors were entered into regression analyses for both of the causal indices in equivalent order to study 1; the additional spatial–temporal measure, DTV, was entered in an extra step ahead of FOL. Analyses for description, prediction, and justification with the same order of entering predictors are presented in Appendix 5.

For *total causal score*, the analysis produced significant Δ*R*^2^ at each stage except for the fifth (Table 4, study 2 regressions). Vocabulary was a significant predictor throughout, with a substantially higher beta than in study 1, although this dropped at each successive stage. Marbles was a significant predictor when it was entered at the second stage, but it dropped out when covariation was added. Covariation was significant and remained so until the inclusion of exponential FOL. Log block design was a significant predictor when added at the fourth stage, and produced a further drop in the beta of marbles. Age became a significant negative predictor at this stage, possibly due to influence of residual variance. When DTV was included, this led to drops in the betas for covariation and log block design, but it was not significant itself. Exponential FOL joined vocabulary and log block design as a positive predictor at the final stage, and the betas for the other predictors dropped further. This suggested that two cognitive ability measures (vocabulary and block design) and one spatial–temporal measure played a significant role in predicting total causal score.

The analysis for *inference* (Table 5, study 2 regression) produced similar outcomes to that for total causal score, except that marbles stayed significant when covariation was entered at the third stage, as in study 1, albeit with a lower beta than there. Both dropped out with the inclusion of log block design. The addition of DTV reduced the beta for all three of these variables, and it was itself significant. FOL was again a significant positive predictor, and its inclusion reduced the betas for the other variables, most notably DTV, which became non-significant.

Overall, the regression analyses revealed clear overlaps between the influence of all the predictors on causal reasoning. In contrast to study 1, covariation was the strongest of the two statistical measures for total causal performance, but marbles nevertheless remained stronger for inference. As in study 1, there were significant differences in marbles score between children giving differing levels of inference response for sinking, *F* = 4.728, *p* = 0.001; absorption, *F* = 2.701, *p* = 0.034; and solution, *F* = 5.371, *p* = 0.001, with *df* = 4, 119 for each, with effects again restricted to differences between those with lower (0, 1, or 2 here) and higher (3 or 4) inference scores. In this study, however, marbles appeared to be more related to non-verbal ability than to the spatial–temporal measures, while covariation was closer to the latter – as if marbles and covariation had swapped status. Neither of the statistical measures survived to the final models in study 2, and their impact was more noticeable here for overall causal performance than for inference.

In contrast to study 1, verbal ability was consistently a strong predictor, alongside non-verbal and FOL in the final model. The impact of the other spatial–temporal measure, DTV, was relatively modest, with FOL substantially reducing the beta of DTV in both analyses. Further regression analysis with all variables confirmed that only FOL was a significant predictor of total causal score (β = 0.356, *p* = 0.000) when both cognitive ability measures – vocabulary and block design – were controlled for.

##### Nature of shared variances between predictors

Factor analysis with varimax rotation, KMO = 0.827, was run as before, to clarify the nature of the shared variances between exp FOL, DTV, log block design, marbles, and covariation. A three-factor model provided the clearest solution, with the first factor explaining 33% of variance, the second 28%, and the third 21% (Table 6, study 2 rotated component matrix). This confirmed log block design as being most closely related to marbles, and FOL to DTV, but covariation as being distinct.

**FIGURE 9:**
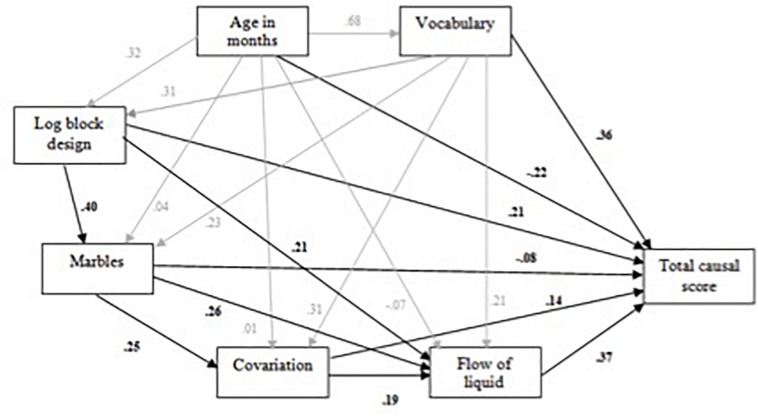
Further extended mediation model for the effects of log block design, covariation, marbles and flow of liquid on overall causal performance (subsidiary effects in gray).

Taking exponential FOL as standing for both spatial–temporal measures, maximum likelihood path analysis was used to examine whether either the extended mediation model identified in study 1 or a reversed version of this (i.e., with covariation and marbles swapping position) provided an adequate fit to the data. These models were contrasted with a further extension of this, in which log block design and marbles fed directly into FOL alongside covariation, reflecting the somewhat more balanced influence of the two statistical measures. For both causal indices, the further extended model provided the best fit to the data: in each case, χ^2^ = 0.315, *p* = 0.575, *df* = 1. Figure 9 illustrates the model plus path coefficients obtained for overall causal performance.

Again, there was a stable pattern of effects in which non-verbal ability, covariation, and probability all support spatial–temporal analysis, but with each also influencing specific aspects of causal thinking directly. In this sample, FOL and vocabulary have the strongest effects for both causal measures: for inference, the effects of FOL and vocabulary are 0.36 and 0.41, and those of non-verbal ability, probability, and covariation, 0.20, 0.01, and 0.09.

As in study 1, further moderation analyses indicated no interaction effect between log block design, marbles, and FOL in predicting causal performance.

### Discussion

Despite differences in the sample, study 2 confirmed the developmental trends observed in study 1 in the ability to analyze probability, covariation, and spatial–temporal information, with if anything clearer increases across the age groups. Once again, the use of all these kinds of information was consistently associated with both overall causal performance and inference, and as before, they appeared to have interrelated influence that was also connected to non-verbal ability.

Marbles was again a significant predictor to begin with in both regression analyses, but its influence dropped with the inclusion of covariation and – in contrast to study 1 – again when block design was added. Covariation was marginally the stronger predictor here and more related to spatial–temporal ability – possibly reflecting the revised measure we used. However, neither statistical measure was a significant predictor in either of the final models, being overtaken in both cases by the spatial–temporal measures, especially FOL. The reduced influence of probability and covariation here is plausibly a reflection of the less developed nature of both competences – and causal inference in this sample. FOL subsumed the influence of DTV, to which it was clearly related, and was the strongest predictor of causal reasoning, alongside verbal and non-verbal ability. The clear impact of verbal ability in this study seems most obviously to be attributable to the more mixed sample, though it cannot in fact be a function of a greater spread of ability, since the variance was actually less than in study 1. Instead, it seems more likely that it reflects vocabulary being a greater influence at lower levels of ability.

In spite of these variations, study 2 largely replicated the results of study 1, while extending them to show that the network of interrelated influences on both spatial–temporal analysis and causal reasoning is based on at least partially unique contributions from probability, covariation, and non-verbal ability. As before, the implication is that statistical and non-verbal ability support spatial–temporal analysis by allowing the capture of patterns of relationship. However, that spatial–temporal ability was a stronger predictor of causal reasoning when statistical and non-verbal ability were less developed and had less direct influence themselves suggests that it is not dependent on these, but rather that each has an independent developmental trajectory – consistent with the factor analysis results.

In line with the results from study 1, the stronger influence of probability on inference and its weak association with covariation suggest that, even in this lower-performing sample, understanding of probability plays a distinctive role in thinking about causal mechanisms, beyond sensitivity to statistical patterns *per se*.

## General Discussion

This study aimed to develop a battery of statistical reasoning tasks, suitable to measure individual differences across a range of developmental levels. It then compared the predictive role of statistical and spatial–temporal analysis in children’s causal thinking about continuous natural processes. Across two studies, in total, five statistical tasks were employed. Taking into account the literature, four of them were developed by the authors for this particular project. Table 7 summarizes the similarities and differences between the two cohorts.

**TABLE 7:** Characteristics of the two cohorts.

	Study 1 (*N* = 107)	Study 2 (*N* = 124)	Significant effects	
			Study 1	Study 2
Hypothesis	Spatial–temporal analysis provides a bridge between observation of continuous processes and their causal analysis			
Sample characteristics	Middle and high SES	Low and middle SES		
Implementation of causal phenomena	A three-stage implementation (prediction, description, explanation)	A five-stage implementation (observation, prediction, justification, testing, explanation)		
Spatial–temporal tasks	Flow of liquid	Flow of liquid	✓	✓
		Distance, time, velocity (DTV)	–	X
Probability and covariation tasks	Randomness	–	X	–
	Marbles	Marbles	X	X
	Cards	–	X	–
	Covariation (computer-based)	Covariation (with cards)	X	X
Verbal task	WASI vocabulary	WASI vocabulary	X	✓
Non-verbal task	WASI block design	WASI block design	✓	✓

### Developmental Trajectories

Children’s responses showed clear progress with age on all tasks. The sample characteristics were different in both studies. Study 1 employed higher SES children, while study 2 employed a mixed SES sample and introduced causal tasks with a scientific method approach alongside a modified covariation task. In study 1, there was greater growth on the causal and spatial–temporal tasks between Y1 and Y3 than between Y3 and Y5; in study 2, there were gains on both across the three age groups. On the causal tasks, in both studies, children’s inference responses were restricted at all ages, and even in Y5, they seemed to find it difficult to explain the mechanisms mediating cause-effect relationships, focusing on more observable and salient factors and variables. In contrast, performance on the liquid flow task in particular approached ceiling by Y5 in both the five- and six-step versions, regardless of sample differences.

Although children showed some different patterns in different tasks, on the present measures, growth in statistical thinking appeared to be slower than that in spatial–temporal ability, but faster than that in causal inference. Past research examining children’s and adult’s covariation and probabilistic thinking in causation – Bayesian and causal learning literature – has focused on the identification of the structure of causal relations between distinct variables (e.g., the relationship between the use of aspirin and headache), or the strength of these (e.g., the degree to which aspirin alleviates headaches; see Lagnado et al., 2007) based on summaries of repeated observations, or compared children’s understanding of common cause and causal chain structures (McCormack et al., 2015, 2016). Although we do not have data on direct comparability with the more detailed tasks used in the developmental literature, our mini-statistics tasks showed sensitivity to differences in children’s statistical thinking that are largely in line with the results from those in the literature.

On the covariation task, children appeared to progress with age in their ability to assess co-occurrences, but to still be refining this further by Y5, especially with respect to numerical quantification. This is in some ways consistent with Shaklee and Mims’ (1981) finding, using a causal event-based approach that children’s strategies for addressing covariation increased in complexity with age. It appeared that in our tasks, older children performed better on the computation of estimates of associational strength. Moreover, although our tasks only focused on co-occurrence (i.e., two cells of the 2 × 2 contingency table), our data also suggest that progress may be slower where the focus is on frequency relationships *per se*, rather than on frequency relations between cause and effect, perhaps because the former are in some sense more abstract.

On both versions of the covariation tasks, one item provided participants with a hundred percent co-occurrence information, which made the interpretation of stimuli unambiguous; the remainder presented incomplete information which increased the ambiguity from 75% in study 2 to 50%, and to 0%. Children mostly seemed to deal well with hundred percent co-occurrence (AB cases, perfect covariation), but less well with the zero correlation, and they had greater difficulty still interpreting degrees of co-occurrence in between with any precision, especially in the youngest age group. This is consistent with Koerber et al.’s (2005) finding that even older children show difficulties in interpreting instances of non-covariation between two distinct events. However, unlike that study, our tasks did not include a conflict between previous beliefs and causal evidence requiring children to test their hypotheses, only to interpret imperfect covariation patterns in a non-causal context. The consistent difficulty in interpreting non-covariation data in both approaches suggests a more fundamental problem that requires further investigation.

In the probability tasks, young children did not show a clear numerical grasp of probability. They did show some understanding of possibilities and their thinking on these tasks seemed to be binary: the majority focused on whether there was a good chance of winning or losing, rather than the degree of that chance. This study therefore agrees with Piaget and Inhelder (1975), White’s (2014), and McCormack et al.’s (2015, 2016) findings, adding to those that thinking with numbers and computing probabilities appeared to start from Y3 onward, consistent with the covariation findings, but in neither case did children begin to approach ceiling performance, even by Y5.

There were also departures from past findings. In particular, many of the younger children in the higher performing study 1 sample did not find it easy to make the distinction between predictable and random events, contrary to Kuzmak and Gelman (1986), with our randomness task showing substantial variation around the mean of 1 (with a high standard deviation, it indicates that majority of children were scoring zero) in Y1, and growth coming predominantly between Y3 and Y5. Conversely, they did not find it harder to deal with frequentist probability task which required sampling (assessed by cards task) neither with other probability task (assessed with marbles), as suggested by work on probability learning (e.g., Brainerd, 1981). The cards task exhibited more or less exactly the same developmental profile as the marbles task – though the use of summary presentations may have helped – and the two were strongly correlated. On both tasks, there was a general improvement of probabilistic thinking through the elementary age range from awareness of variation in likelihood to numerically precise calculation of this. While recent cognitive-developmental work has focused on tasks sensitive even to the youngest children’s level of skill (Schlottmann and Wilkening, 2011), our tasks stretched even the oldest children in the sample, as intended, especially with respect to proportional calculations – ratios and decimals – as opposed to more basic judgments.

### Relationships Between Causal Reasoning, Spatial–Temporal, Covariation, and Probabilistic Thinking

Despite the covariation and probability tasks drawing on related types of frequency information, a distinction between our key predictors was confirmed by both correlational and factor analyses in both studies. In study 1, frequentist cards and other probability tasks (marbles) were closely related as compared with covariation and randomness tasks, and the covariation task appeared to demand a more distinct competence. In study 2, both spatial–temporal measures (liquid flow and DTV) loaded onto the same factor, while marbles and covariation again remained independent, albeit with marbles being associated with block design, once more suggesting that the ability to utilize covariation, probability, and spatial–temporal information requires different competences.

Most people would agree that forms of statistical reasoning, as emphasized by Humean approaches to causality, are useful for causal thinking about discrete events, which lend themselves easily to frequency-based analyses, but the present study showed that this form of thinking also relates to causal thinking about extended processes, which have no perceptually distinct components. One basic possibility is that the role of statistical sensitivity and non-verbal ability is primarily one of enabling forms of pattern detection. Block design assesses the ability to analyze and reconstruct perceptual patterns, which facilitates the detection and representation of causal effects; covariation assesses the ability to track connections, which facilitates the identification of relationships between variables and outcomes; and probability assesses the ability to track the “definiteness” of outcomes, which facilitates awareness of strength of effect, e.g., the relative impact of a variable, in this case on speed of effect. The integration of Kantian mechanism-based and Humean statistical thinking highlighted by our results echoes recent theoretical debates in the literature on causal thinking about discrete events (see Waldmann, 2017). However, our novel contribution is not just the application to continuous processes, but to the correlational, individual difference approach. The same approach might also be useful in the future to study how children’s thinking about discrete causal events develops.

While the statistical variables were largely not significant predictors in the regressions, the path analyses, however, found that probability and covariation formed a network of interrelated competences influencing causal reasoning, along with non-verbal and verbal ability. It should be noted that regression models portray the relations from the raw data and cannot provide more sensitive statistics as to, for instance, what is the nature of residuals after each step. We can observe the effects of each variable by assessing the change in beta values after each step. Thus, even if a variable remains non-significant in the final model, the chance in the beta values shows us whether the variable contributed to the model one way or another. In both studies, we captured these widespread interactions with the path analyses. It is the nature of this network that needs to be explained.

Naturally, our interpretations are limited to the task characteristics and statistical methods. We cannot conclude whether or not the basic understanding of, for instance, randomness assists causal inference in continuous processes. Similarly, it is not clear why marbles was a stronger predictor than covariation in both studies. Although we have an idea about the possibilities drawing marbles to lose its predictive power for both causal indices in study 2 (e.g., we had a more powerful covariation task, and there was an additional spatial–temporal variable involved, which reduced the variance explained by both marbles and covariation), these do not analyze the unique nature of the tasks. Moreover, differences between the samples in terms of relative developmental level across the various indices may have played a role. That the spatial–temporal predictors trumped statistical predictors fits with the notion that temporal information overrides covariation when a causal structure needs to be inferred, as in the event literature with adults (see also Waldmann and Holyoak, 1992; Cheng, 1993; Waldmann et al., 1995; Cheng et al., 1996) and with children (see Siegler, 1975; Mendelson and Schulz, 1976; Bullock et al., 1982). Although our focus is on continuity rather than contiguity, the data from both studies show similar outcomes: statistical thinking appears to be promising in terms of supporting reasoning about mechanisms.

Understanding of probability seems to do something more than is captured by this proposed indirect influence on causal thinking. The relationship of performance on the marbles task in both studies to inference of mechanisms and its more distinct predictive power, especially in the higher performing study 1 sample, both suggest that it promotes some other additional insight. A cross-check between probability and causal task performance shows that in both studies children who had perfect marbles scores were more likely to provide high-level inference scores and make reference to mechanisms (*n* = 21 in study 1; *n* = 19 in study 2). Probabilistic thinking seems therefore to be important not only for the identification of the strength of the effects of variables, but for considering unseen elements of causal processes. It is plausible that awareness of probability drives a general heightening of sensitivity to the operation of unseen factors, as argued in the introduction. This would be consistent with children exhibiting similar limitations in probability scores and references to mechanism.

However, construction of a dynamic mental representation tying spatial–temporal information together to envisage the operation of *specific* mechanisms still requires in addition a time-based analytical and constructive ability, as captured by the FOL tasks. In other words, non-verbal ability, probability, and covariation help by enabling children to identify variables and to sense that there is more to be explained about how these operate, but as the data suggest, it remains primarily spatial–temporal ability that takes them beyond this to coordination of actual information and ideas of mechanism.

Verbal ability also appears to be necessary to get all of this off the ground, given its influence among the lower-performing sample in study 2. However, all these competences seem to have distinct developmental trajectories and converge on support of causal inference. The nature of the growth of this convergence – and how far it can and possibly needs to be deliberately promoted – requires further investigation.

Two lines of inquiry can investigate this convergence between causal reasoning and distinct competences, one empirical, e.g., experimental studies aiming to elaborate on the aspects of statistical and spatial–temporal thinking, and another methodological, e.g., studies investigating the nature of the causal tasks in relation to intelligence tests. Regarding the first line of inquiry, the present study provided the first dataset, using intelligence measures as controls. It should be noted that these measures have high statistical reliability and explanatory power, and they challenge substantially other tasks present in the same model. One can expect statistical form of thinking to be more predictive in different models when intelligence measures are excluded. In fact, when we did so, the covariation task explained a unique variance in causal thinking (β = 0.178, *p* = 0.034) along with age and FOL. The increase in beta values of flow liquid was also substantial. This highlights the above interpretation taking into account the nature of shared variances in models, i.e., how the strongest predictor subsumes the beta of others in regression models.

In line with the methodological inquiry, a follow-up study employed three intelligence measures and investigated their relevance to the above causal tasks. The data were analyzed based on the Tucker-Drob (2009) model, which is constructed based on an integration of Horn–Cattell’s theory of fluid and crystallized intelligence (Horn and Cattell, 1966). The study found very high correlations between the measures, where general intelligence factor explained about 62% of the variance in causal tasks. This effect was independent of age and the model was able to analyze the nature of the residuals (Dündar-Coecke, under review). This result suggests that there may also be a strong link between spatial–temporal reasoning and intelligence types, which clearly merits further investigations.

## Conclusion

An important contributor to causal reasoning about continuous processes is spatial–temporal analysis. When its influence is compared with that of statistical reasoning, it remains as the strongest predictor. However, statistical reasoning made both direct contributions and exerted an indirect influence *via* spatial–temporal analysis. The findings here highlight the multiple and complex determinants involved in such thinking. This is the first investigation employing process-based causal tasks to examine the role of covariation and probability alongside spatial–temporal ability. Further studies can explore the unique nature of the tasks and their relations to other forms of reasoning.

## Data Availability Statement

All datasets generated for this study are included in the article/Supplementary Material.

## Ethics Statement

Ethical approval for the studies involved human participants was obtained from the UCL Institute of Education Research Ethics Committee, University College London. Children’s verbal responses were also received before the start of each session. Children were not included in testing without their verbal consents even if their parental consent was available.

## Author Contributions

SD-C conceptualized and developed the idea, conducted the research and analyses, and wrote the original manuscript. AT supervised these processes, verified the analytical processes, and reviewed and edited the manuscript. He was also involved in data and reliability analyses. AS supervised these processes and reviewed and edited the manuscript. All authors discussed the results and contributed to the final manuscript.

## Conflict of Interest

The authors declare that the research was conducted in the absence of any commercial or financial relationships that could be construed as a potential conflict of interest.
