# Supplementary material for: The Development of Spatial–Temporal, Probability, and Covariation Information to Infer Continuous Causal Processes
Source: Front Psychol. 2021 Mar 5;12:525195. doi: 10.3389/fpsyg.2021.525195 (PMC7973365; doi:10.3389/fpsyg.2021.525195)
Supplement: Supplementary file 1 [file Data_Sheet_1.docx]

Appendices

**Appendix 1. Causal task scripts**

For *sinking*, children saw a stone and a blueberry of similar size but different densities, which sank at different rates in a half-meter tall large transparent jar of still water. Children needed to predict outcomes ahead of witnessing simultaneous demonstration of the two instances, then to describe e.g. the rates of sinking, and to explain the outcomes, as a measure of causal inference assessing identification of basic factors (e.g. softness/hardness/heaviness of the materials), operative variables (e.g. relative weight of the materials) and mechanisms (e.g. object density, the role of water). A typical testing session involved the following scripts:

Experimenter: “I have got these two objects here: a stone and a blueberry. Do you want to hold them?” (A pause) “I am going to drop them in the water. What do you think will happen?”

Child: (predictions)

Experimenter: “Do you think that the same thing will happen to both objects?”

Child: (predictions)

Experimenter: “Okay. Thank you. Please watch carefully now. I will drop the objects and you will watch.” The experimenter drops the items together at the same time and asks: “Did you notice anything, what?”

Child: (descriptions)

Experimenter: “Did the same thing happen to both objects?”

Child: (descriptions)

Experimenter: “Okay thank you. Why do you think things happened that way?”

Child: (explanations)

Experimenter: “Do you think there might be another reason for that?”

For *absorption*, children saw water rising from a petrie dish through strips of tissue and blotting paper of the same length/width, the water rising faster through the more open structure of the tissue. Similar to sinking protocol, children needed to predict outcomes ahead of witnessing simultaneous demonstration of the two instances, then to describe e.g. the rates of absorption, and to explain the outcomes, as a measure of causal inference assessing identification of basic factors (e.g. softness/hardness of the materials), operative variables (e.g. relative softness/hardness/porousness of the materials) and mechanisms (e.g. relative size of the holes allowing water to rise up). A typical testing session involved the following scripts:

Experimenter: “I have got these two strips of paper here: a tissue and a blotting paper. Do you want to touch them?” (A pause) “I am going to dip them in the water. What do you think will happen?”

Child: (predictions)

Experimenter: “Do you think that the same thing will happen to both papers?”

Child: (predictions)

Experimenter: “Okay. Thank you. Please watch carefully now. I will dip the papers and you will watch.” The experimenter dips the end of the papers in water together at the same time and asks: “Did you notice anything, what?”

Child: (descriptions)

Experimenter: “Did the same thing happen to both papers?”

Child: (descriptions)

Experimenter: “Okay thank you. Why do you think things happened that way?”

Child: (explanations)

Experimenter: “Do you think there might be another reason for that?”

For *solution*, children saw the same small quantities of table and rock salt dissolve in warm water. The small quantity of the salt was assured with two equally very small spoons. The greater surface area to volume of the table salt led to more rapid solution. Similar to sinking and absorption protocols, children needed to predict outcomes ahead of witnessing simultaneous demonstration of the two instances, then to describe e.g. the rates of absorption, and to explain the outcomes, as a measure of causal inference assessing identification of basic factors (e.g. softness/hardness of the materials), operative variables (e.g. relative softness/hardness/compactness of the materials) and mechanisms (e.g. relative size of the grains allowing water to penetrate in). A typical testing session involved the following scripts:

Experimenter: “I have got these two kinds of salts here: table salt and rock salt. I am going to drop a tiny piece from each in the water. What do you think will happen?”

Child: (predictions)

Experimenter: “Do you think that the same thing will happen to both kinds of salt?”

Child: (predictions)

Experimenter: “Okay. Thank you. Please watch carefully now. I will drop the salts and you will watch.” The experimenter drops the samples of the salts together at the same time and asks: “Did you notice anything, what?”

Child: (descriptions)

Experimenter: “Did the same thing happen to both kinds of salt?”

Child: (descriptions)

Experimenter: “Okay thank you. Why do you think things happened that way?”

Child: (explanations)

Experimenter: “Do you think there might be another reason for that?”

**Appendix 2. Causal tasks scoring system**

Each task had the same three-stage structure, in which children: (1) inspected the contrasting materials and were asked what they thought would happen when they were put into the water (prediction from *prior knowledge*); (2) watched the focal events and were asked to *describe* what they had noticed; (3) were asked to *explain* why they thought things had happened in the way that they had seen. At each stage, they were encouraged to give as full an answer as they could. Observation duration was longer for dissolving due to the salt types took some reasonable amount of time to dissolve. Observation duration was shorter for sinking, and moderate for absorption.

Data from these tasks were used to compute three types of measures: (1) components: individual total scores for accurate prediction from *prior knowledge*, *description, and explanation* across three tasks, (2) *composite* scores for each task (sinking, absorption, solution), and (3) *a total causal score* combining these.

(1) Components: children’s responses for prior knowledge, description and explanation computed across three tasks (see Table 12 for scoring system). Prior knowledge and description were scored for accuracy of anticipating / reporting differences in sinking/absorption/solution rate (1 point per object. Therefore the scores were between 0-6 for both prior knowledge and description). Explanation scoring began at the minimal level of the observed factor(s) (score of 1); via making explicit that these are variables linked to the observed differences in speed of the contrasting examples (score of 2); to a statement about the underlying mechanism which produced the effect (score of 3). Therefore the scores were between 0-9) across the three tasks.

(2) For the composite measure, children’s responses for each phenomenon scored independently The minimum and maximum score for each phenomenon varied from 0 to 7.

(3) For the total causal score all these scores were combined (0-21; alpha = .751). The total number of *mechanism* level explanation responses were also noted across the tasks (0-3), as a separate measure of higher causal thinking. Table 12 provides a detailed version of the scoring system.

Table 12. Scoring system for causal tasks

| Component | Sinking | Absorption | Solution |
| --- | --- | --- | --- |
| Prediction from prior knowledge  (0-2) | Correct prediction for stone (i.e. sinks) =1  Correct prediction for difference between stone and berry (i.e. sink at different speeds)=1 | Correct prediction for tissue paper=1  Correct prediction for difference between tissue and blotting  paper =1 | Correct prediction for table salt=1  Correct prediction for difference between table and rock salt=1 |
| Description of observation  (0-2) | Correct description for stone=1  Correct description for berry=1 | Correct description for tissue paper=1  Correct description for blotting paper=1 | Correct description for table salt=1  Correct description for rock salt=1 |
| Explanation/  inference  (0-3) | No/irrelevant explanation=0  Weight/size without difference between objects=1  Weight/size with difference=2  Density and mechanism=3 | No/irrelevant explanation=0  Thickness/softness/texture etc. without difference between types of paper=1  Thickness/softness/texture etc. with difference=2  Nature of papers/holes and mechanism=3 | No/irrelevant explanation=0  Grain/size etc. without difference between types of salt=1  Grain/size etc. with difference=2  Grain/size etc. with surface area and mechanism=3 |

To confirm reliability, two researchers subsequently scored all responses independently from the audio-recordings. Agreement was 93%, and final scores were assigned following discussion and checking the audios in the small number of instances where there was a difference (example responses are shown in the Table 13).

Table 13. Examples of explanation responses

| Phenomena | Level 1 | Level 2 | Level 3 |
| --- | --- | --- | --- |
| Sinking | “They are heavy and they sank to the bottom” | ‘The stone is heavier than the berry so they sank to the bottom differently” | “They are both heavier than the water and cannot hold air in it so they sank to the bottom. But the stone sank quicker than the berry because it’s got more stuff in it so the water can’t hold it up as it did to berry.” |
| Absorption | “If you dip the paper in the water they get wet because they’re soft” | “The tissue paper is thinner than the other paper so water rises faster in it” | “The tissue paper has holes in it that help water to rise up. Water holds on the walls of the holes and layers and that helps it to climb up. Other paper has some space in it, but not as much as the tissue paper.” |
| Solution | “They go into water because they’re small and spread out” | “The table salt is smaller than the rock salt so it disappears quicker.” | “The size of the two types of salt is different. And this is more rocky so water cannot go into it easily. They both dissolve in the water, but rocky one takes more time than the table salt.” |

**Appendix 3. Scientific method (Study 2) causal task scripts**

For *sinking* children saw a stone and a grape of similar size and colour but different densities, which sank at different rates in a half meter tall large transparent jar of still water. The five-stage design implemented by the following scripts:

*Description of observation:* “I've got these two objects here; a stone and a grape. I’m going to drop them in the water. Please watch carefully.” Did you notice anything, what?

*Prediction:* “Now, I will show you these three objects, marble, playdough ball, cherry tomato. Can you rank their sinking order/rate, which one will sink fastest and which one will sink slowest? Which one will be in the middle?”

*Judgment*: “Why did you rank/order them in that way?”

*Testing*: “Could you test these to see if your prediction is correct?” “Was your prediction correct?”

*Explanation:* “Why do you think things happened that way?” “Do you think there might be another reason for that?”

For *absorption*, children saw water rising from a petrie dish through the strips of tissue and blotting paper of the same length/width, the water rising faster through the more open structure of the tissue. Similar to sinking protocol, the five-stage design implemented by the following scripts:

*Description of observation:* “I've got these two strips of paper here; a tissue paper and a blotting paper. I’m going to dip them in the water. Please watch carefully.” Did you notice anything, what?”

*Prediction:* “Now, I will show you these three objects, a piece of fabric, a piece of cardboard, and a piece of foam. Can you rank them, which one will soak up water fastest and more and which one will soak up slowest? Which one will be in the middle?”

*Judgment*: “Why did you rank/order them in that way?”

*Testing*: “Could you test these to see if your prediction is correct?” “Was your prediction correct?”

*Explanation:* “Why do you think things happened that way?” “Do you think there might be another reason for that?”

For *solution*, children saw the same small quantities of table and rock salt dissolve in warm water. The small quantity of the salt was assured with two equally very small spoons. The greater surface area to volume of the table salt led to more rapid solution. Similar to sinking and absorption protocols, the five-stage design implemented by the following scripts:

*Description of observation:* “I've got two types of salt here; some table salt and some rocky salt. I’m going to put them in the water. Please watch carefully.” Did you notice anything, what?”

*Prediction:* “Now, I will show you these three objects, muscovado sugar, demerara sugar, and caster sugar. Can you rank their disappearing time, which one will disappear fastest, which one will be the slowest, and which one will be in the middle?”

*Judgment*: “Why did you rank/order them in that way?”

*Testing*: “Could you test these to see if your prediction is correct?” “Was your prediction correct?”

*Explanation:* “Why do you think things happened that way?” “Do you think there might be another reason for that?”

**Appendix 4. Scientific method (study 2) scoring system**

Scores for children’s description, prediction, justification, and explanation responses were arrived at in the same way as the previous study. Using the modified system shown in the Table 14, composite measures were computed for each response component as follows:

0-3 for *description*

0-9 for *prediction* and *justification*

0-12 for *explanation*

0-33 for *total causal score* (alpha = .724).

0-3 for the number of *mechanism* level responses made by children.

Table 14. Scoring system for causal task

|  | Sinking | Absorption | Solution |
| --- | --- | --- | --- |
| Description of observation  (0-1) | No observation=0  Observing different  sinking rate=1 | No observation=0  Observing different rate of water rising=1 | No observation=0  Observing different solution rate=1 |
| Prediction following observation  (0-3) | Any prediction  plasticine comes first=0  Any prediction tomato comes first=1  Marble-tomato-plasticine=2  Marble-plasticine-tomato=3 | Any prediction foam comes first=0  Fabric-foam-cardboard=1  Cardboard-fabric-foam=2  Fabric-cardboard-  foam=3 | Any prediction  other than below=0  All same=1  Caster-demerara-muscavado=2  Caster-muscavao-demerara=3 |
| Justification of predicted order  (0-3) | No/irrelevant explanation=0  Only weight=1a  Only size=1b  Both without coordination=2  Both with  coordination=3 | No/irrelevant explanation=0  Only thickness=1a  Only softness=1b  Both without coordination=2  Both with  coordination=3 | No/irrelevant explanation=0  Only material=1a  Only size=1b  Surface area or compactness=2  Both coordinated=3 |
| Explanation (abstraction of causal factor; link to speed difference i.e. variable; coordination of variables; mention of how variable affects speed) (0-4) | No/irrelevant explanation=0  Weight/size without difference=1  Weight/size with difference=2  Density (weight/size coordinated)=3  Density with  mechanism=4 | No/irrelevant explanation=0  Thickness/softness  without difference=1  Both with difference=2  Structure/holes coordinated)=3  Optimum hole size  with mechanism=4 | No/irrelevant explanation=0  Grain/size etc.  without difference=1  Grain/size etc. with difference=2  Grain/size etc.  coordinated with compactness=3  Surface area, compactness and solvent mechanism =4 |

Example explanation responses illustrating the additional level of scoring for coordination of causal variables are also shown in Table 15.

Table 15. Level 3 and 4 explanation responses

| Phenomena | Level 3 | Level 4 |
| --- | --- | --- |
| Sinking | “The marble is smaller, heavier and harder than the playdough and the tomato, and the stone is like the marble.” | “The marble and the stone are dense, the water doesn’t have enough strength to make them float, and they are denser than the others that is why they sank faster.” |
| Absorption | “The tissue paper and the fabric are lighter and have texture. They have room, which allows water to rise up.” | “The tissue paper has bigger layers and holes which allows water go into it faster then the blotting paper. The cardboard has holes too but they’re bigger, and it’s harder for the water to spread through them. More compact more difficult for water to rise” |
| Solution | “The table salt is less compact like caster sugar, they are both smaller and softer, and easier to spread around.” | “The table salt and caster sugar has less surface area, the water can cover it in no time and break it all down. For the bigger chunks it takes longer for the water to get around it and into it, so it takes longer to dissolve. Some materials (e.g. demerara) have harder walls, compactness.” |
